# Trophic niche breadth of pond zooplankton species using stable isotope analysis and the relationship with the abiotic and biotic factors

**DOI:** 10.1098/rsos.180917

**Published:** 2019-01-16

**Authors:** Hideyuki Doi, Kwang-Hyeon Chang, Shin-ichi Nakano

**Affiliations:** 1 LAFWEDY, Faculty of Agriculture, Ehime University, 3-5-7, Tarumi, Matsuyama, Ehime, Japan; 2 Graduate School of Simulation Studies, University of Hyogo, 7-1-28 Minatojima-minamimachi, Chuo-ku, Kobe, Japan; 3 Department of Environmental Science and Engineering, Kyung Hee University, Seocheon-dong, Giheung-gu, Yongin-si, Korea; 4 Center for Ecological Research, Kyoto University, 2-509-3 Hirano, Otsu, Shiga, Japan

**Keywords:** food web, stable isotopes, niche space, *Daphnia*, copepod

## Abstract

Zooplankton species have different feeding habits, but the diversity of their food resources and the factors governing them are still largely unknown. We here estimated the differences in the trophic niche breadths of dominant zooplankton species in ponds, using stable isotopes. To understand the differences in trophic niches of different zooplankton species, we measured the carbon and nitrogen stable isotope ratios and calculated the nearest-neighbour distance (NND), and standard deviation of NND (SDNND) of the bi-plot space of stable carbon and nitrogen isotopes in pond zooplankton. We tested the relationship between the NND/SDNND and environmental factors, as well as the zooplankton biomass, using generalized linear models (GLMs). For cladocerans, including *Bosmina, Ceriodaphnia* and *Daphnia,* the NNDs were significantly correlated with the biomass, pond morphology (volume and depth), total phosphorous (TP) and fish presence. For copepod species, including *Eodiaptomus* and cyclopoids, NNDs were significantly correlated with pond morphology, TP and fish presence, but not with biomass. In GLMs of SDNND, significant correlated factors were less than those for NND, and for some species, pond morphology and TP were significantly correlated with SDNND. Here, we found that the NND and SDNND of zooplankton species were related to various factors, including their biomass, predator presence, pond size and water quality. For cladocerans, biomass may be supported by trophic niche breadth, probably because of the consequences of resource competition. Also, predation and ecosystem size may influence trophic niche breadth due to changes in zooplankton behaviours.

## 1. Introduction

Understanding the factors driving trophic niche breadth is a central question in ecology [1–3] and can have important implications for the transfer of energy to higher trophic levels [3]. The trophic niche breadth of the predators, such as fish species, has been evaluated in previous studies [3–7], because the trophic niche breadth of predators may strongly affect food-web structures [2,3]. Although primary consumers have an important role in determining the food-web structure, especially in energy and material transfer to higher trophic levels [1,8], the trophic niche breadth of primary consumers has not been evaluated thoroughly.

In aquatic systems, zooplankton species are primary and secondary consumers and a key part of aquatic food webs [8,9]. It is well established that in lakes and ponds, the composition of zooplankton communities is determined by the availability of food [10–12]. The large calanoid copepods and cladocerans are especially competitive for food resources [12,13]. Therefore, ponds and their zooplankton populations can provide model ecosystems for investigating the trophic niche breadth of primary consumer communities.

Stable carbon and nitrogen isotopes ratios (δ^13^C and δ^15^N) reflect food sources and trophic positions of consumers, respectively, and the ratios between these isotopes have been widely used to analyse food webs in natural systems [14,15]. Stable isotope techniques have been used to correlate carbon–nitrogen bi-plots and niche space among species to provide an estimate of trophic niches [6,16–20]. In the present study, we measured the isotope niches from the bi-plot space of stable carbon and nitrogen isotopes in different zooplankton species to estimate differences in trophic niches among the different species in a community.

In this study, we estimated the differences in the trophic niche breadths of dominant zooplankton species in ponds, using isotope niche space derived from stable isotope bi-plot space. We also tested the relationships between isotope niche indices, the nearest-neighbour distance (NND) and standard deviation of NND (SDNND), of zooplankton species and environmental factors, including phytoplankton biomass (chlorophyll *a*), total phosphorus, pond morphology (volume and depth) and the presence of zooplanktivorous fish and their biomass in the study ponds, to investigate the driving factors of zooplankton trophic niche breadths in these ponds.

## 2. Material and methods

### 2.1. Study sites

This study was conducted in 14 ponds in Matsuyama, Japan (33°48′-50′ N, 132°48′-55′ E). The region is temperate and has distinct seasonal temperatures. Pond volumes ranged from 850 to 54 400 m^3^ and mean depths ranged from 2.0 to 7.1 m [21]. These ponds experienced an invasion of largemouth bass, introduced in the 1970s–1980s, and zooplanktivorous fishes are mainly bluegill and small Crucian carp. We sampled the central regions of the 14 ponds from 27 October to 9 November 2005. We had the pond owners' permission to collect the plankton.

### 2.2. Collection and preparation of zooplankton

Zooplankton were collected by vertical net towing (200-μm mesh net) from near the bottom to the surface in each pond. For identification and enumeration, the collected zooplankton were preserved in approximately 5% formalin. The body lengths of individuals were measured, and their biomass was calculated using length–weight regression [22]. For stable isotope analysis, fresh zooplankton samples were maintained in a cooler during transport to the laboratory.

### 2.3. Stable isotope analysis

We collected the following dominant zooplankton: *Daphnia* spp. (mainly *Daphnia galeata*, eight collection sites), *Eodiaptomus* copepods (mainly adults, 11 sites), *Bosmina* spp. (mainly *Bosmina longirostris*, 13 sites), *Ceriodaphnia* spp. (10 sites) and cyclopoids (mainly adults, 13 sites). Zooplankton individuals were sorted and placed in a tip cup (approx. 10–100 individuals per sample for each species), and examined under a stereomicroscope. All zooplankton samples were dried at 60°C for 48 h and stored in desiccators until the isotope ratios were analysed. The carbon and nitrogen isotope ratios of the samples (*n* = 3) were measured using a continuous-flow isotope mass spectrometer (Integra CN, Sercon Co., UK). All isotopic data are reported using the conventional δ notation, where δ^13^C or δ^15^N = (*R*_sample_/*R*_standard_ − 1) (/‰). *R* is the ^13^C/^12^C or ^15^N/^14^N ratio for δ^13^C or δ^15^N, respectively. Vienna Pee Dee Belemnite and N_2_ in air were used as international standards for δ^13^C and δ^15^N, respectively. The overall analytical error values were within ±0.2 (/‰) for both δ^13^C and δ^15^N.

### 2.4. Analysis of NND

We analysed the distance between trophic niches using the NND of the zooplankton species on the basis of their mean δ^13^C and δ^15^N values (electronic supplementary material, table S1). To compare niche differences among the 14 ponds, we considered the potential isotope space of the zooplankton competitors and standardized NND, according to the method described by Layman *et al.* [17] and Jackson *et al*. [18]. Thus, we calculated NND as the nearest distance to a competitor per potential niche space of δ^13^C and δ^15^N bi-plots. First, we calculated the Euclidean distance (ED) between the isotope values of zooplankton species in δ^13^C and δ^15^N bi-plot spaces using the datasets in electronic supplementary material, appendix S1 and the following equation:

2.1 $$ED_{i}=\sqrt{{\left(\delta^{13}C_{i}-\delta^{13}C_{j}\right)}^{2}+{\left(\delta^{15}N_{i}-\delta^{15}N_{j}\right)}^{2}},$$

where *i* and *j* indicate species *i* and *j*, respectively.

Second, we calculated the isotopic ellipses of each pond using the following equation with 95% confidential interval [18], because there were limited points for calculating convex hull in the isotope bi-plots (*n* = 5):

Finally, we calculated NND*_i_* as the smallest ED*_i_* among combinations of species *i* and other zooplankton species per PNS of each pond:

2.2 $$\mathrm{NN}D_{i}=\frac{\mathrm{smallest}\;ED_{i}}{\mathrm{the}\;\mathrm{isotopic}\;\mathrm{ellipses}\;\mathrm{of}\;\mathrm{the}\;\mathrm{pond}}.$$

NND*_i_* indicates the distance of the trophic niche of species *i* from the other species in the zooplankton community; smaller NND values suggest higher trophic redundancy for the species. The carbon isotope ratios of the zooplankton varied within the individual ponds (electronic supplementary material, appendix S1). Thus, the NND primarily showed the carbon isotope differences between species. Layman *et al.* [17] suggested a possible limitation of trophic niche estimation (i.e. NND) using stable isotopes. Temporal and spatial variations in isotope values of primary producers contribute to variations in the isotope values of consumers. Zooplankton have short isotope turnover times (typically one to two weeks; [23,24]). Thus, we assumed that this potential limitation was relatively small for the zooplankton community. We also calculated SDNND (Standard deviation of NND, [17]) to estimate the variations in NND.

### 2.5. Measurement of biotic and abiotic factors

To determine chlorophyll *a* concentration, we collected 2 l of surface water (depth, 0–50 cm) with a column sampler. A 250-ml aliquot of each water sample was filtered through a 0.2-µm Nuclepore filter (Millipore Co. Billerica, MA, USA) to retain the seston. Each filter was placed in a glass test tube, and *N*,*N*-dimethylformamide was added to extract chlorophyll *a.* Chlorophyll *a* concentration was determined using a fluorometer (10-AU; Turner Designs, Sunnyvale, CA, USA). We measured the pH of the surface water using Twin-pH portable meters (Horiba Co. Tokyo, Japan). Total phosphorus (TP) concentration of the surface water was determined by colorimetric analysis with a continuous-flow system (AutoAnalyzer 3, BRAN + LUEBBE, Norderstedt, Germany). The presence and the absence of plankton-feeding fish, including *Carassius* spp., bluegill sunfish and small carp, were estimated based on observations from the shore and angling. We used the presence–absence data for plankton-feeding fish to define the presence or the absence of fish predators of zooplankton.

### 2.6. Statistical analysis

To determine the differences in isotopic niche indices (NND and SDNND) among the zooplankton species in the ponds, we performed one-way analysis of variance (ANOVA). To analyse the relationships between biomass and other parameters, including the NND of the species, we used a generalized linear model (GLM). We used zooplankton biomass, chlorophyll *a* of surface water, mean water depth of the pond, total pond volume, TP of surface water and zooplanktivorous predator presence (as categorized data) as the explanatory variables in the model. Preliminary Shapiro–Wilk tests for normality showed that TP and chlorophyll *a* were not normally distributed; thus, we transformed these variables using a log_10_ (*x* + 1) transformation. We also selected the best model of GLMs using a backward stepwise procedure based on Akaike Information Criterion (AIC). The environmental factors and the biomass of zooplankton used in the GLM are shown in electronic supplementary material, tables S2 and S3. In all statistical analysis, we set *α* = 0.05. We performed all statistical analysis using the R software v. 3.3.2 [25].

## 3. Results

### 3.1. NND and SDNND of zooplankton

**Figure 1.**
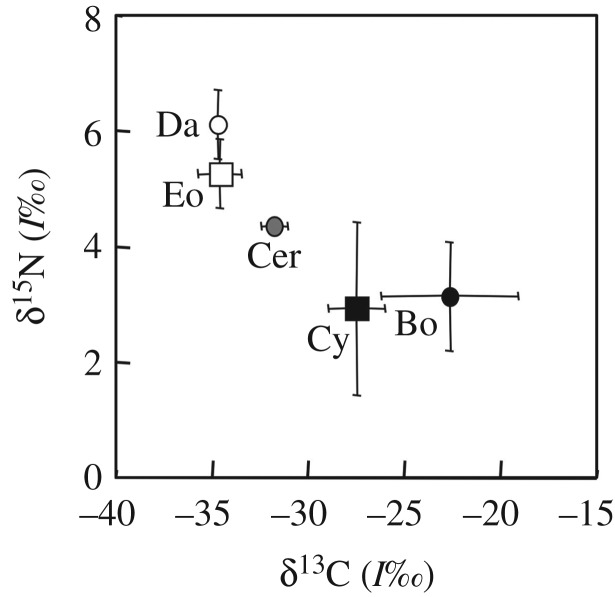
Bi-plot space of carbon and nitrogen isotopes of zooplankton in two of 14 ponds. Bo, Cer, Cy, Da and Eo indicate *Bosmina, Ceriodaphnia,* Cyclopoids*, Daphnia* and *Eodiaptomus* spp., respectively. The error bars indicate the standard deviation of mean.

**Figure 2.**
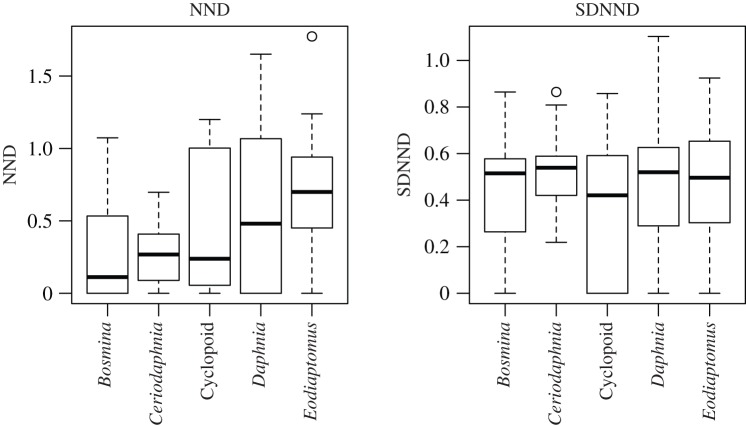
Boxplots for NND and SDNND of zooplankton species in all ponds. The boxes indicate ± 25% quartiles with the median (bar), and the bars indicate ± 1.5*x* quartiles. The points are outliers.

The NND and SDNND calculated by the isotope bi-plot space in each pond ecosystem are shown in figure 1 and are provided as raw data i.e. electronic supplementary material, table S1. Figure 2 shows the differences in isotope values among zooplankton species in the ponds, which had almost all dominant species. The NND and SDNND were not significantly different among the zooplankton species (figure 2, ANOVA, *F* = 2.01, *p* = 0.090 for NND, *F* = 0.720, *p* = 0.581 for SDNND). Higher variations in NND and SDNND among the ponds were observed in all species; for example, NNDs of *Daphnia* and *Eodiaptomus* varied fivefold among ponds.

### 3.2. Relationships between the NND/SDNND and environmental factors

**Figure 3.**
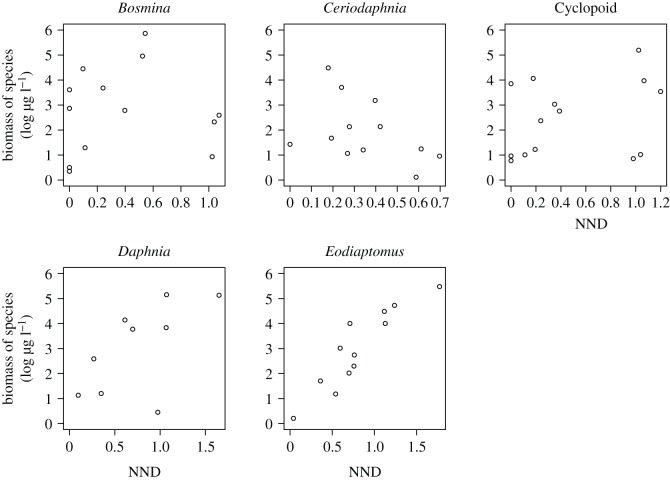
Relationships between NND and the biomass of zooplankton species; NND was calculated from the bi-plot space of carbon and nitrogen isotopes of zooplankton in the ponds.

The best GLM results showed that the biomass of *Bosmina, Ceriodaphnia* and *Daphnia* were significantly related to NND (table 1*a* and figure 3). Values of NND were significantly affected by their biomass in these zooplankton species, although other factors, especially fish presence, pond volume and TP also were significantly related to the NNDs. By contrast, the biomass of copepod species, *Eodiaptomus* and cyclopoids, were not significantly related to their NNDs. For both species, fish presence and TP were significantly related to their NNDs.

**Table 1.** The full and best generalized linear models between (*a*) NND and (*b*) SDNND) and the explanatory factors. In the models, NND, Depth, Chl *a* and TP indicate NND, mean depth of pond, chlorophyll *a* and total phosphorus of surface water, respectively. Italic font indicates significant factors (*p* < 0.05).

	*Daphnia*		*Eodiaptomus*		*Bosmina*		*Ceriodaphnia*		Cyclopoids	
	FULL	BEST	FULL	BEST	FULL	BEST	FULL	BEST	FULL	BEST
(*a*) NND										
biomass	0.389	*0*.*400*	0.308		−1.954	*−1*.*768*	−0.690	*−0*.*884*	0.186	
Chl *a*	−0.005	−0.005	−0.014	−0.014	−0.032	−0.027	−0.002		−0.006	
fish	−0.654		0.957	*1*.*055*	−3.124	*−2*.*997*	−0.182		−3.131	*−3*.*035*
volume	0.878	*1*.*076*	0.664	1.198	1.794	1.477	−0.912		1.707	
depth	−0.152	−0.192	0.168		−0.122		0.175		−0.323	
TP	0.147		2.684	*2*.*809*	0.296		0.176		−3.672	*−2*.*959*
(intercept)	−2.633		−3.870	−5.094	0.266	0.645	5.154	*2*.*549*	4.225	*8*.*604*
AIC	−25.64	−26.17	12.630	9.360	7.040	3.440	*0*.*300*	*−6*.*140*	11.910	8.000
*Δ*AIC		−5.100		−3.270		−3.600		−6.440		−3.910
*R*^2^	0.769	*0*.*8188*	0.532	*0*.*628*	0.389	*0*.*681*	0.141	*0*.*299*	0.452	*0*.*640*
(*b*) SDNND										
biomass	0.854	0.648	−0.240		0.741	0.723	0.558	0.774	0.216	
Chl *a*	−0.012	−0.014	0.001		−0.006	−0.006	−0.011	−0.013	−0.006	
fish	0.559		1.185	0.895	−0.884	−0.852	−0.340		−0.349	
volume	*3*.*905*	*4*.*060*	−0.436		−0.565	−0.609	0.052		1.889	*1*.*741*
depth	−1.036	*−1*.*129*	0.168		−0.385	−0.375	0.023		−0.599	*−0*.*558*
TP	−1.425	−1.501	1.627	*1*.*180*	−0.092		0.065		−0.287	
(intercept)	−7.176	−6.859	0.808	0.524	*5*.*834*	*5*.*845*	2.202	*2*.*333*	−2.242	−2.397
AIC	1.730	0.380	−0.330	−6.080	−5.000	−6.950	6.190	−1.160	3.600	−1.980
*Δ*AIC		−1.350		−5.750		−1.950		−7.350		−5.580
*R*^2^	0.442	0.526	0.151	0.382	0.538	*0*.*613*	0.141	0.201	0.115	*0*.*330*

For SDNND, the relationships with the factors assessed were almost not significant (table 1*b*). For *Daphnia* and cyclopoids, lake morphology, pond volume and water depth were significantly related to the SDNNDs. For *Eodiaptomus,* the SDNND was significantly related to TP.

## 4. Discussion

Our results showed that there was high variation in the NNDs, and SDNNDs of zooplankton species among ponds. From the generalized linear models, we identified some factors that potentially drive the variation in NND and SDNND. For the cladocerans, including *Bosmina, Ceriodaphnia* and *Daphnia,* species biomass in the ponds was one of the potential drivers of isotopic niche breadth, indicating that biomass may be related to the differences in trophic niches in populations sharing limited food resources, such as different algal species. However, these cladocerans are filter feeders that feed on phytoplankton, bacteria and fungi [11,12,26]. *Daphnia, Ceriodaphnia* and *Bosmina* have different body size, and their feeding efficiencies can differ according to their feeding ranges and filtering rates as well as by size, motility and morphology of available prey items [27]. Thus, the phenomenon might result from the different feeding fractions as a consequence of competition for resources in their populations. In fact, the positive and negative correlations were mixed among species. We speculate that this was a consequence of competition among cladocerans.

By contrast, for copepod species, including *Eodiaptomus* and cyclopoids, NNDs were not significantly related to biomass, probably because competition for their food resources was lower than that in the cladocerans. The feeding mode of copepod species is more predacious, and they feed mainly on larger algae and microzooplankton such as small protozoan, rotifers and cladocerans often with feeding selectivity [26,28,29]. The differences in their feeding habits would induce the differences in the correlation of NND with their biomass. Further study using laboratory or field experiments controlling the biomass and resources is needed to reveal the causality of this phenomenon.

For most zooplankton species, NNDs were significantly correlated with fish presence, but that of *Daphnia* significantly correlated with the pond volume. Pond volume may be indirectly related to NND, due to ecosystem-size effects on population dynamics, food-chain length and increased species diversity [30]. The presence of zooplanktivorous fishes has a strong impact on zooplankton populations [31]. Zooplankton species often change their behaviours, such as daily vertical migration [32], and consequently change their food sources [33,34]. Thus, fish presence may be an important factor causing zooplankton to alter their feeding modes and consequently drive the trophic niche breadth of zooplankton species in these ponds. Here, we only used the presence data of zooplanktivorous fishes, so further study is needed to test this hypothesis by comparing zooplankton with the biomass or abundance of zooplanktivorous fishes.

For copepod species, including *Eodiaptomus* and cyclopoids, their NNDs and SDNND were significantly correlated with TP. Total phosphorous is classically an index of pond primary productivity [31], so the primary production may be indirectly related to trophic niche breadth of the predatory species, probably due to the trophic-cascade effect from primary producers.

In the SDNNDs, the significantly related factors were less than those for NND, and for some species, pond morphology and TP were significantly related to SDNND. These relations for SDNNDs may also be related to factors discussed above, for NND, such as ecosystem-size effect and pond primary productivity. However, for SDNND, plankton biomass and fish presence were not significant factors for all species. The differences in NND and SDNND results may be caused by the different meanings of the indices. The NND showed more variation in primary food resources than SDNND because the NNDs of the species were primarily determined by carbon isotope variations in isotope bi-plot space, whereas SDNND showed more variation in the total area of niche space. The different behaviours in the isotopic niche positions may allow us to estimate trophic niche space based on different aspects.

Although the trophic breadth and the plasticity in the zooplankton species may also be evolutionarily driven [8], the present study found that variations in trophic breadth in the ponds ranged across a region. Therefore, variation in trophic breadth of zooplankton species would be mainly driven by current environmental factors and interspecific competition, and may be indirectly driven by their evolutionary history.

Our study had a few limitations. We only estimated the dominant zooplankton competitors. We could not consider interactions between rare species and different trophic guilds, such as rotifers and ciliates, because it is difficult to measure their isotopic signatures. Previous studies have suggested strong competition among mesozooplankton because their food ranges often overlap [11] while microzooplankton including rotifers and ciliates are rather bacterivores [23]; therefore, our results may reflect relationships between trophic niches and their abundances, although we did not estimate all possible interactions. When applying indices of niche differences in future studies, closer attention should be paid to interactions with species in which stable isotopes cannot be easily measured. Although lifespans of zooplankton species are short [23,31], we only estimated competitors within a season. Competition and environmental factors during other seasons could also affect relationships between trophic niches and the biomass of competitors. Although our study evaluated trophic niche breadth and potential drivers of the dominant zooplankton species in nature, further studies are required to estimate the seasonal dynamics of trophic niche competition and the consequent biomass of competitors. Also, in this study, we calculated the NND and SDNND using the raw isotope data, however, the abiotic effect, such as anthropogenic N input, also influence the isotope values of the organisms. So, using the isotope niche space, we should further consider such abiotic effects on the calculations.

In conclusion, we showed the trophic niche breadths of zooplankton species in the habitats even within narrow areas. We also found that factors related to the trophic niche breadth were variable, including their biomass, ecosystem size, predator presence, as well as total phosphorus. Our study data limits us from making conclusions about general trends in these dynamics, but this is an initial step towards the evaluation of trophic niche breadth of primary and secondary consumers in natural habitats, using stable isotope techniques.

## Supplementary Material

Supplemental Materials

## Data accessibility

Our data are deposited in the Dryad Digital Repository: http://dx.doi.org/10.5061/dryad.6jr5797.
