# Supplementary material for: Trophic niche breadth of pond zooplankton species using stable isotope analysis and the relationship with the abiotic and biotic factors
Source: R Soc Open Sci. 2019 Jan 16;6(1):180917. doi: 10.1098/rsos.180917 (PMC6366219; doi:10.1098/rsos.180917)
Supplement: Supplemental Materials [file rsos180917supp1.pdf]

Supplemental Materials

Table S1. Carbon ($\delta^{13}C$, ‰) and nitrogen ($\delta^{15}N$, ‰) isotopes of dominant zooplankton species used to calculate the NND of species.

Site	*Daphnia*		*Eodiaptomus*		*Bosmina*		*Ceriodaphnia*		Cyclopoids	
	$\delta^{13}C$	$\delta^{15}N$	$\delta^{13}C$	$\delta^{15}N$	$\delta^{13}C$	$\delta^{15}N$	$\delta^{13}C$	$\delta^{15}N$	$\delta^{13}C$	$\delta^{15}N$
1			–27.0	5.6	–24.3	4.0	–28.1	7.9	–27.6	7.2
2			–36.4	3.9	–29.6	5.6	–30.4	5.3	–32.5	4.6
3	–18.4	6.1	–25.1	3.3	–22.3	5.1	–20.5	5.5	–17.5	5.6
4	–25.1	5.6	–23.8	5.4			–30.7	6.7		
5	–36.7	5.4	–29.9	8.7	–31.7	6.2	–30.6	6.2	–25.0	6.0
6	–29.7	6.6	–37.8	7.6	–27.8	8.1	–29.0	5.8	–29.6	6.3
7					–29.5	6.2	–31.4	5.6	–26.5	8.9
8	–31.2	6.3			–24.3	4.1	–29.1	4.5	–29.0	4.6
9					–25.3	5.5	–27.8	4.0	–25.2	5.0
10	–34.7	6.1	–34.6	5.3	–22.7	3.1	–31.8	7.4	–27.5	2.9
11	–30.1	8.5	–28.8	9.3	–29.3	3.6			–26.5	6.6
12			–22.4	8.1	–23.9	5.5			–22.4	5.3
13			–15.6	5.8	–22.1	3.8			–20.7	4.6
14	–38.7	3.6	–36.6	7.4	–36.2	4.6	–38.6	4.7	–34.8	9.1

Table S2. Environmental factors of the 14 ponds used in this study. Log TP and Chl-a indicate log-transformed total phosphorus and chlorophyll *a* of surface water, respectively.

Sites	pH	Water depth (m)	Log TP ($\mu g\ L^{-1}$)	Log Chl-a ($\mu g\ L^{-1}$)
1	6.5	4.7	1.5	1.3
2	7.1	5.0	1.1	2.0
3	9.6	3.3	2.0	2.0
4	8.0	6.5	1.9	1.2
5	7.6	2.5	1.1	1.7
6	7.1	5.0	2.7	1.3
7	7.3	2.8	1.2	1.1
8	7.6	2.0	1.2	0.4
9	9.0	1.5	1.4	1.6
10	7.6	7.1	1.1	0.6
11	7.3	4.1	1.7	1.7
12	8.2	3.2	1.3	1.6
13	9.6	3.0	1.2	1.7
14	8.3	2.8	1.2	1.3

Table S3 Biomass (μg L^{-1}) of dominant zooplankton species. “Fish” indicates presence (P) or absence (0) of plankton-feeding fish in the pond.

Site	Fish	*Daphnia*	*Eodiaptomus*	*Bosmina*	*Ceriodaphnia*	Cyclopoids
1	P	0.0	53.7	36.0	4.3	2.4
2	P	0.0	40.5	16.6	7.4	14.8
3	0	2.3	53.7	15.2	23.2	19.7
4	0	0.6	111.8	0.0	2.3	0.0
5	0	171.8	0.0	38.7	39.7	33.5
6	0	168.3	238.6	351.2	87.9	57.3
7	P	0.1	0.0	84.6	7.5	1.3
8	0	61.9	0.2	1.5	2.5	1.6
9	P	0.0	0.0	2.6	0.1	1.7
10	0	42.6	9.0	9.2	1.6	1.8
11	0	45.3	140.5	12.4	3.2	52.2
12	P	0.0	109.4	0.4	0.0	46.1
13	P	0.0	87.6	0.7	0.0	1.2
14	0	12.3	20.3	141.4	1.9	179.5
